# Monitoring Lipophilic Toxins in Seawater Using Dispersive Liquid—Liquid Microextraction and Liquid Chromatography with Triple Quadrupole Mass Spectrometry

**DOI:** 10.3390/toxins13010057

**Published:** 2021-01-13

**Authors:** Ainhoa Oller-Ruiz, Natalia Campillo, Manuel Hernández-Córdoba, Javier Gilabert, Pilar Viñas

**Affiliations:** 1 Department of Analytical Chemistry, Faculty of Chemistry, Regional Campus of International Excellence “Campus Mare Nostrum”, University of Murcia, E-30100 Murcia, Spain; ainhoa.oller@um.es (A.O.-R.); ncampi@um.es (N.C.); hcordoba@um.es (M.H.-C.); 2 Department of Chemical and Environmental Engineering, Regional Campus of International Excellence “Campus Mare Nostrum”, Polytechnic University of Cartagena, E-30203 Cartagena, Spain; javier.gilabert@upct.es

**Keywords:** lipophilic marine toxins, seawater, dispersive liquid–liquid microextraction, liquid chromatography, triple quadrupole tandem mass spectrometry

## Abstract

The use of dispersive liquid–liquid microextraction (DLLME) is proposed for the preconcentration of thirteen lipophilic marine toxins in seawater samples. For this purpose, 0.5 mL of methanol and 440 µL of chloroform were injected into 12 mL of sample. The enriched organic phase, once evaporated and reconstituted in methanol, was analyzed by reversed-phase liquid chromatography with triple-quadrupole tandem mass spectrometry. A central composite design multivariate method was used to optimize the interrelated parameters affecting DLLME efficiency. The absence of any matrix effect in the samples allowed them to be quantified against aqueous standards. The optimized procedure was validated by recovery studies, which provided values in the 82–123% range. The detection limits varied between 0.2 and 5.7 ng L^−1^, depending on the analyte, and the intraday precision values were in the 0.1–7.5% range in terms of relative standard deviation. Ten water samples taken from different points of the Mar Menor lagoon were analyzed and were found to be free of the studied toxins.

## 1. Introduction

The importance of the role of photosynthetic microorganisms in the functioning of aquatic ecosystems is indisputable; however, their ability to produce toxins means that these ecosystems must be monitored, with the ultimate aim of preserving animal and human health [1]. Aquatic toxins can appear in both fresh and saltwater systems. Marine toxins are secondary metabolites generated by various phytoplankton species subjected to adverse climatic and environmental conditions and are also called phycotoxins. Since phytoplankton serves as food for many aquatic organisms, biomagnification phenomena may occur if toxins enter the food chain, causing serious health problems in humans [2]. Filter feeder organisms, which may filter large volumes of water during their lives, are the species in which the greatest bioaccumulation of marine toxins has been detected.

When the episodes of phytoplankton blooms occur in areas where shellfish are cultured or areas for fisheries, or even in bathing waters, they can lead to environmental, health, and economic problems. In recent years, there have been several cases of phytoplankton proliferation along the coasts of Europe [3], particularly in intensive shellfish culture areas, including Spain (e.g., Andalusia [4], Galicia [5], the Valencia Community [6], and Murcia [7]). The increase in water temperature, changes in salinity, and water dissolved nutrients stoichiometry in coastal waters, mainly due to agricultural run-off or urban wastewater discharges, are some of the factors suggested for an increase in the proliferation of potentially toxic phytoplankton, thus increasing the concentration of toxins in aquatic environments [8]. Changes in these variables have been crucial for setting up the problems detected in recent years in the waters of the Mar Menor lagoon, especially in the summer of 2016, as a result of a severe eutrophication process. Since then, monitoring programs, including toxins, were activated. The lagoon is located in the Southwestern Mediterranean Sea and is the largest hypersaline in Europe with 135 Km^2^ and 4 m average depth on the Southeast coast of Spain.

Depending on their polarity, marine toxins can be classified as hydrophilic, lipophilic, or amphiphilic [9]. The main compounds included in the lipophilic group are okadaic acid (OA) and dinophysitoxins (DTXs), pectenotoxins, yessotoxins, azaspiracids (AZAs), and cyclic imines, the last mentioned including spirolides (SPXs), pinnatoxins, pteriatoxins, and gymnodimins. According to the symptoms observed in animal studies or human poisoning, aquatic toxins can be classified into those that cause paralytic, amnesic, diarrhetic, neurotoxic shellfish poisoning, and ciguatera fish poisoning. Although additional syndromes are increasingly appearing, each type of poisoning is associated with a specific group of biotoxins [10].

The reference analytical methods for the European Union are laid down in the 2019 Regulation (No. 2019/627) [11] with liquid chromatography and tandem mass spectrometry (LC-MS/MS) for controlling lipophilic toxins. Thus, the literature describes different methodologies for the separation, identification, and quantitation of marine toxins, with LC being the most widely used, especially in combination with a fluorescence detector (FLD) [12,13] and diode array detector (DAD) [14] for the analysis of shellfish and fish, or a mass spectrometer (MS) [2,14,15,16,17,18,19,20,21,22,23,24,25,26,27,28,29,30,31,32,33,34,35,36,37,38] to check compliance with the maximum permitted levels in shellfish and fish, or sediments [2,14,15,16,17,18,19,20,21,22,23,24,25,26,27,28,29,30,31,32,33,34,35,36,37,38] and waters [22,23,24,26,27,28,29,31]. In fact, the high sensitivity and selectivity of LC with MS/MS makes this technique the best choice for the simultaneous evaluation of different lipophilic biotoxins [39].

While high levels of these toxins may be found in bivalves due to their continuous filtration of water, trace levels can be expected in seawater. Therefore, a preconcentration step is required before any instrumental analytical measurement. The conventional solid phase extraction (SPE) technique has been widely applied for the analysis of water [22,26,27,28] and seafood after a solid–liquid extraction step [14,15,19]. SPE provides the simultaneous cleanup of the sample and has demonstrated high levels of robustness and reliability; however, SPE entails long analysis times and a high consumption of both organic solvents and samples. Despite the proven advantages of miniaturized methods, very few applications can be found in the literature for the lipophilic toxins studied here. Dispersive micro solid phase extraction (DMSPE) [23] and magnetic solid phase microextraction (MSPE) [30] have been applied for analyzing seawater and shellfish, respectively. A miniaturized procedure based on ionic liquid dispersive liquid–liquid microextraction (IL-DLLME) [31] has been used to preconcentrate three cyanotoxins (microcystin RR, microcystin LR, and nodularin) and two phycotoxins (domoic acid and OA) from algae-based food supplements, OA being the only common analyte with the presented method here. Moreover, a DLLME procedure based on conventional solvents is presented. As regards the detection system, MS/MS based on triple quadrupole (QqQ) is used here, while the hybridation of simple quadrupole and time of flight was coupled to the LC for the determination of cyano- and phycotoxins [31]. 

**Figure 1:**
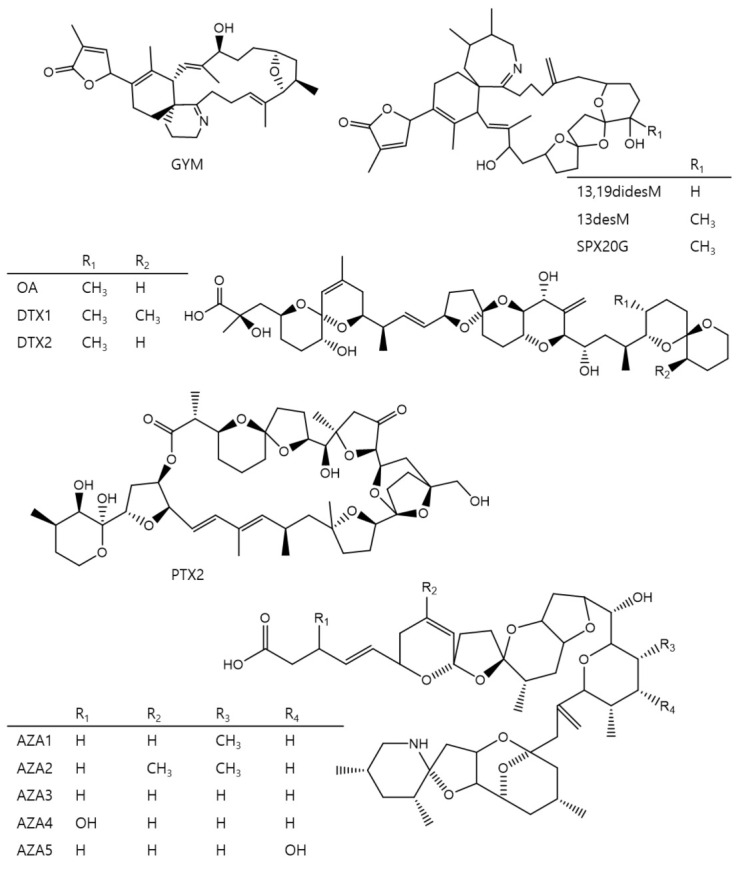
Molecular structures of the monitored toxins.

The present work describes a novel method based on DLLME for the determination of thirteen lipophilic toxins, which chemical structures are shown in Figure 1, gymnodimine (GYM), 13,19-didesmethyl spirolide C (13,19-didesM), 13-desmethyl spirolide C (13-desM), 20-spirolide G (SPX20G), OA, dinophysitoxin 1 (DTX1), dinophysitoxin 2 (DTX2), pectenotoxin 2 (PTX2), azaspiracid 1 (AZA1), AZA2, AZA3, AZA4, and AZA5 in seawater. The preconcentrated extracts are analyzed by LC-MS/MS with QqQ mass analyzer.

## 2. Results and Discussion

### 2.1. Chromatographic Separation and MS Conditions

Because of the lipophilic nature of the toxins studied, LC was applied in the reversed phase (RP) mode using a C18 stationary phase. The isocratic elution mode was not suitable for separating the thirteen compounds in a reasonable time, so different gradients were tested using different mixtures of 2 mM ammonium acetate and 0.1% formic acid (FA) solutions prepared in water (solvent A) and methanol (MeOH) (solvent B) [29,37]. Under the conditions finally selected, the elution was initiated with 25% of solvent B at a flow rate of 0.3 mL min^−1^, increased to 60% in 3 min, and maintained for 5 min, which allowed the elution of GYM and the two desmethyl-SPXs. Then, the percentage of the organic solution was increased to 75% in 0.5 min and maintained for 6.5 min to elute SPX20G, OA, DTX2, PTX2, and AZA4. In the next stage of the gradient, the flow rate was increased to 0.4 mL min^−1^ in 0.5 min in proportion, followed by another increase of solvent B to 85% in 4.5 min, which allowed the elution of DTX1 and the four AZAs still retained. Finally, as a cleaning stage, 95% of solvent B was programmed, and the initial conditions were restored using a flow of 0.5 mL min^−1^, as shown in Table 1. For high proportions of MeOH in the mobile phase, the pressure in the column decreased so that the mobile phase flow rate could be increased, and the analysis time shortened. The optimization of the mobile phase composition was also assayed in the absence of salt or acid, and no significant differences in the resolution of the peaks were observed, while the absence of acid and/or salt in the mobile phase led to a decrease in the ionization efficiency of the compounds.

The parameters of the MS detector were optimized in several steps, working first in full-scan mode from 80 to 1000 amu (*m/z*) to identify the precursor ion of each compound. All the toxins showed greater sensitivity in positive ionization mode at the Electrospray Ionization (ESI) source. Then, different fragmentation voltages and collision energies were applied, thus generating different product ions. The two most sensitive Multiple Reaction Monitoring (MRM) transitions were selected for each analyte, except GYM, for which only one transition was located (Table 2). The identification of each toxin was based on its retention time and the different MRM transitions involving the formation of product ions with the highest *m/z* values.

The influence of other variables in the ESI source were studied: temperature (200–350 °C), gas flow (4–12 L min^−1^), nebulizer pressure (20–50 psi), and capillary voltage (2000–5000 V). The conditions finally adopted were 350 °C, 8 L min^−1^ nitrogen flow rate, 40 psi, and 4500 V. Even though the pairs of analytes OA/DTX2 and AZA4/AZA5 showed the same precursor ions and even the same MRM transitions, this did not pose a problem for their quantitation since they eluted at different retention times.

### 2.2. Optimization of the DLLME Procedure

The first experiments were directed towards selecting the extractant phase, for which eleven different solvents were tested, some heavier (CCl_4_, CHCl_3_, CH_2_Cl_2_, 1,2-dichloroethane, and 1,1,2,2-tetrachloroethene) and some lighter (methyl isobutyl ketone (MIBK), 2-octanol, 1-undecanol, 1-dodecanol, 2-octanone, and 2-undecanone) than water. An aqueous solution containing 1 ng mL^−1^ of one analyte from each of the four families studied (13,19didesM, OA, AZA1, and PTX2) was used to simplify the study. By means of a micro syringe, a mixture containing 1.5 mL MeOH and 440 µL extractant was injected into 8 mL of the standard solution, and the mixture was manually shaken for a few seconds before centrifuging for 3 min at 855× *g*. The enriched extracts obtained from solvents of lower density than water were directly injected (15 μL) into the LC system, while, in the case of solvents of higher density than water, the extract was evaporated and reconstituted in 150 µL MeOH before injection.

**Figure 2:**
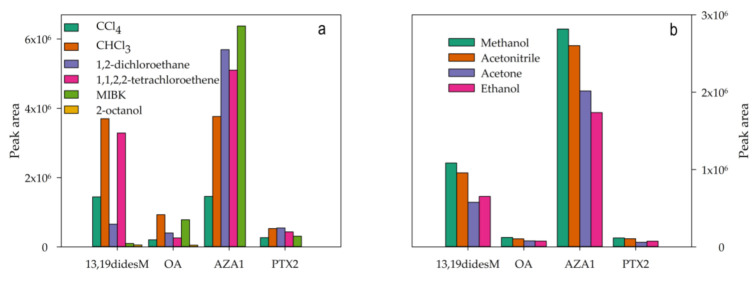
Influence of the nature of the: (**a**) extractant phase and (**b**) dispersant solvent on dispersive liquid–liquid microextraction (DLLME) microextraction efficiency. 13,19-didesM: 13,19-didesmethyl spirolide C; OA: okadaic acid; AZA1: azaspiracid 1; PTX2: pectenotoxin 2.

Except for MIBK, the lighter-than-water solvents did not preconcentrate the toxins (Figure 2a). Chloroform provided the highest efficiency for 13,19didesM and OA, while no significant differences were found between CHCl_3_ and 1,2-dichloroethane for PTX2. Considering that AZA1 was more efficiently extracted in MIBK, but recovery with CHCl_3_ was better, the latter solvent was chosen. Thus, peak areas in the absence of preconcentration were increased by factors of between 300 and 910, which corresponded to PTX2 and 13,19-didesM, respectively, when chloroform was used as the extractant solvent.

Acetone, ethanol, acetonitrile (AcN) and MeOH were tested as dispersant solvents, and although no great differences in sensitivity were found for OA and PTX2, MeOH provided better results for 13,19didesM and AZA1 (Figure 2b) and was, therefore, selected.

**Figure 3:**
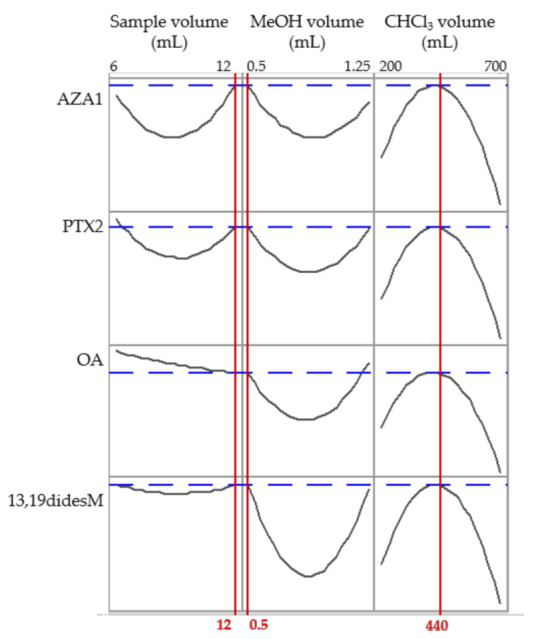
Response plots showing the influence of sample, dispersant, and extractant volumes on the relative response of the compounds. 13,19-didesM: 13,19-didesmethyl spirolide C; OA: okadaic acid; AZA1: azaspiracid 1; PTX2: pectenotoxin 2.

The volumes of the three components of the DLLME ternary mixture were studied simultaneously using a central composite design (CCD; α = 0.5; 8 cubic points; 6 axial points and 6 central points). The aqueous phase volume was studied in the 6–12 mL range, the dispersant from 0.5 to 2 mL, and the extractant from 200 to 700 µL, making a total of 20 runs, whose combinations are shown in Table 3. The results obtained were fitted to a response surface by quadratic polynomial regression. Figure 3 shows peak area values for each toxin selected from each group, normalized with respect to their average area in the corresponding set of experiments. The experimental matrices (Table 3) were generated, and the obtained results were evaluated using the Minitab 19.0 statistical package. Sensitivity for all compounds increased as the volume of CHCl_3_ increased up to 440 μL, and then decreased for higher values, probably due to a dilution effect (Figure 3). On the other hand, generally, the highest and the lowest volumes assayed for sample and dispersant phases, respectively, provided the best results. Thus, the conditions finally selected corresponded to 12 mL of water sample, 0.5 mL of MeOH, and 440 μL of CHCl_3_.

When the pH of the sample was varied between 4 and 9 by adding FA or ammonia, an acid pH was found to favor the extraction of toxins of acidic nature (PTX2, DTXs, and OA), whereas the basic ones, such as AZAs, SPXs, and GYM, showed higher extraction efficiencies in alkaline media, as expected. Consequently, a compromise was adopted, and the approximately neutral pH of the samples was not modified.

### 2.3. Validation of the Procedure and Matrix Effect

The procedure developed was validated in terms of precision, limits of detection (LODs) and quantitation (LOQs), linearity range, and accuracy. Calibration graphs were obtained by least-squares linear regression analysis, representing the chromatographic peak area vs. the concentration of each compound at seven concentration levels in triplicate. In all cases, the regression coefficients (R^2^) were greater than 0.998 for the ranges shown in Table 4.

To assess the possible existence of a matrix effect, the slopes of the aqueous standard calibration graphs were compared with those obtained by applying the standard additions method to five seawater samples obtained close to and far from the coast. No significant differences at the 95% confidence level were detected when applying the ANOVA test (as described in the Section 4) for each toxin separately because the “*p*” values obtained were higher than 0.05 for all the compounds. Consequently, the absence of a matrix effect was confirmed, and quantitation of the samples was carried out using aqueous standard solutions.

LOD and LOQ values were calculated, considering the analyte concentrations that provided analytical signals 3 and 10 times higher than the noise, respectively. The LODs were between 0.2 and 5.7 ng·L^−1^, and the LOQs in the 0.7–19 ng·L^−1^ range (Table 4). The precision of the method was studied in ten consecutive analyses of seawater samples fortified at 50 ng·L^−1^. The relative standard deviation (RSD) values were found to be between 0.1 and 7.5%, which corresponded to GYM and DTX1, respectively (Table 4). These RSD values demonstrated the very good repeatability of the developed procedure.

The accuracy of the procedure was checked in recovery studies. Two samples, taken at 1 m from the surface in the surf area in a beach and one sample collected 25 m off the coast, were fortified at two concentration levels, 10 and 50 ng·L^−1^, for all compounds except DTX1, for which concentrations of 50 and 100 ng·L^−1^ were used. The recoveries obtained are shown in Table 5, which were in the 82–123% and 90–121% ranges for the lowest and the highest fortification levels, respectively.

Table 6 shows a comparison between the DLLME-LC-QqQ-MS/MS procedure and others previously published for the determination of lipophilic toxins in seawater using LC-MS. Note that although lower LODs were achieved with some of those procedures that used SPE [22,26,27,28], these conventional sample treatments involved longer times (up to 9 h) and a much greater consumption of sample (between 200 and 500 mL). In contrast, the proposed DLLME procedure requires considerably lower volumes of organic solvent and sample, as well as a shorter application time (around 5 min). The DMSPE procedure developed by Zhang et al. [23] takes a similar time to that presented here with lower LODs, which may be attributed to the highly sensitive Q Exactive MS detector used, as in another SPE-based approach [28]. Nevertheless, the disadvantages inherent in using extractant solid dispersed phases compared with dispersed liquid phases should be considered. Moreover, the number of toxins that can be determined using the procedure studied here is higher than that mentioned in the above studies.

### 2.4. Analysis of Seawater Samples

The DLLME-LC-QqQ-MS/MS procedure was used to analyze ten seawater samples collected from the Mar Menor lagoon. None of the samples contained the studied toxins, at least above their corresponding LODs.

**Figure 4:**
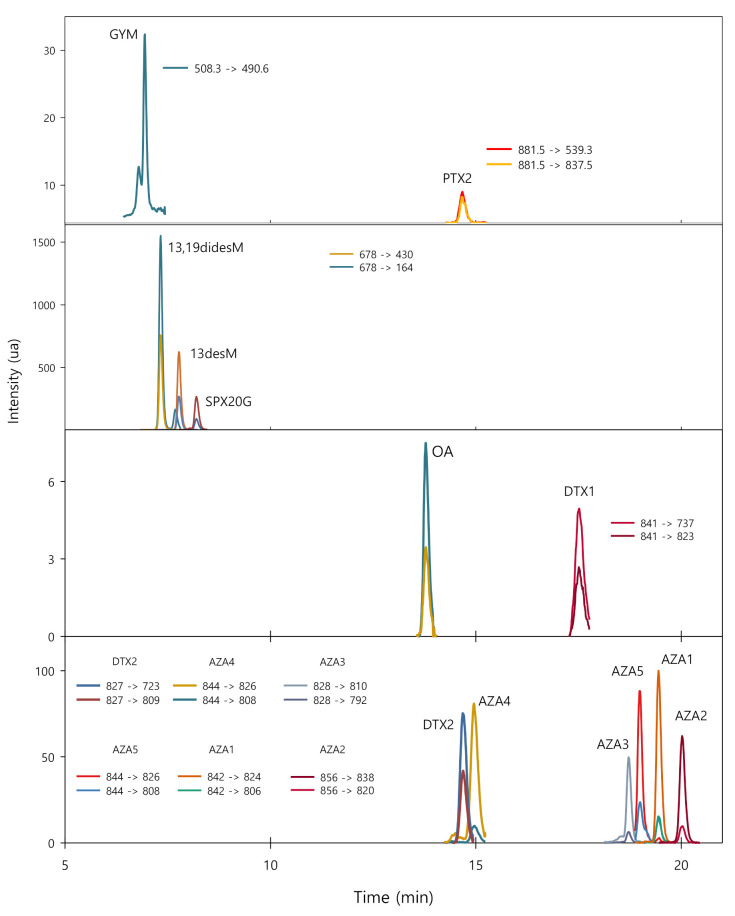
Extracted ion chromatograms (EICs) obtained using the DLLME with LC-QqQ-MS/MS procedure for a sample fortified at 10 ng L^−1^ (50 ng L^−1^ for DTX1). GYM: gymnodimine; 13,19-didesM: 13,19-didesmethyl spirolide C; 13-desM: 13-desmethyl spirolide C; SPX20G: 20-spirolide G; OA: okadaic acid; DTX: dinophysitoxin; PTX2: pectenotoxin 2; AZA: azaspiracid.

Figure 4 shows the extracted ion chromatograms (EICs) obtained for a seawater sample fortified with the analytes studied at 10 ng·L^−1^, except in the case of DTX1, which was fortified at 50 ng·L^−1^. For each toxin, the selected transitions are specified. No interfering peaks were observed at the different retention times for the toxins, demonstrating the good selectivity of the proposed procedure. The analytes were identified from their retention times, the transitions provided by their mass spectra and comparing the percentage of each transition obtained for standard solutions, and unfortified and fortified samples.

## 3. Conclusions

The combination of DLLME with LC-QqQ-MS/MS allowed for the sensitive and selective analysis of seawater samples. For the first time, a high number of lipophilic marine toxins, belonging to four chemical families, has been efficiently preconcentrated under the green analytical chemistry guidelines, achieving high analysis speed, great efficiency, low operational costs since the consumption of organic toxic solvents and sample volume are very low, and an environmentally friendly analytical procedure. The absence of any matrix effect allowed quantitation of the samples against aqueous standards. None of the studied toxins was detected in the waters collected from the Mar Menor lagoon in samples collected in 2019.

## 4. Materials and Methods 

### 4.1. Reagents

The standards 13,19didesM (7.06 ± 0.24 µg·mL^−1^), 13desM (7.23 ± 0.10 µg·mL^−1^), SPX20G (7.01 ± 0.61 µg·mL^−1^), OA (16 ± 0.8 µg µg·mL^−1^), DTX1 (6.40 ± 0.33 µg·mL^−1^), DTX2 (2.01 ± 0.11 µg·mL^−1^), AZA1 (1.08 ± 0.06 µg·mL^−1^), AZA2 (1.05 ± 0.08 µg·mL^−1^), AZA3 (1.03 ± 0.07 µg·mL^−1^), AZA4 (1.01 ± 0.03 µg mL^−1^), and AZA5 (1.09 ± 0.03 µg·mL^−1^) were acquired from Cifga, S.A. (Lugo, Spain) in individual ampoules containing 0.5 mL of a methanolic solution at the specified concentrations. GYM (2.50 ± 0.13 µg mL^−1^) and PTX2 (4.40 ± 0.13 µg mL^−1^) were supplied by the Institute for Marine Biosciences of the National Research Council Canada (NRC CNRC, Halifax, Canada). The individual standard solutions were stored at −18 °C, and the working standard solutions were prepared daily in methanol (MeOH) and stored at 4 °C.

The MeOH used for the mobile phase was LC-MS grade. The rest of the employed solvents were ACS-grade. Acetonitrile (AcN), acetone, and ethanol were obtained from Chem-Lab NV (Zedelgem, Belgium) and chloroform, carbon tetrachloride, dichloromethane, 1,2-dichloroethane, 1,1,2,2-tetrachloroethene, methyl isobutyl ketone (MIBK), 1-octanol, 1-undecanol, 1-dodecanol, 2-octanone, and 2-undecanone from Sigma–Aldrich (Steinheim, Germany). Formic acid (FA, 98%) and ammonium acetate were purchased from Panreac (Barcelona, Spain). The purified water was obtained through a Milli-Q system (Millipore, Bedford, MA, USA).

### 4.2. Instrumentation

The chromatographic system consisted of a 1200 UHPLC apparatus from Agilent (Waldbronn, Germany) provided with a quaternary pump (G1312A) and a Zorbax SB-C18 reversed phase column (Agilent, 75 × 2.1 mm, 3.5 µm) thermostated at 30 °C. The samples were injected into the LC system (15 μL) by means of an automatic sampler using 2 mL amber vials provided with 250 µL micro-inserts with polymeric feet. The mobile phase was composed of an aqueous solution of 2 mM ammonium acetate and 0.1% FA (solvent A) and a methanolic solution of 2 mM ammonium acetate and 0.1% FA (solvent B) working in elution gradient mode. Table 1 shows the gradient program applied.

The analytes were detected by using a triple quadrupole mass spectrometer (Agilent, G6410A), equipped with an electronic impact ionization (ESI) source operating in positive mode, applying the following parameter values: pressure of nebulizer gas (nitrogen), 40 psi; capillary voltage, 4500 V; temperature and flow of the drying gas, 350 °C, and 8 L min^−1^, respectively. The mass spectra were analyzed in the range *m/z* 80–1000 amu. Data acquisition and method development were performed using the software “Agilent Mass Hunter Data Acquisition” (Qualitative Analysis and Quantitative Analysis, Agilent (Waldbronn, Germany)). The multiple reaction monitoring mode (MRM) was used. Minitab 19 software program was used for statistical evaluation of the results. For statistical evaluation of a possible matrix effect in the samples, a one way ANOVA test was used to compare two means from two independent groups, the slopes obtained by standard calibration and the slopes from standard additions to the samples, using the F-distribution. The null hypothesis for the test is that the two means are equal. Therefore, a significant result means that the two means are unequal.

Individual solutions (1 µg mL^−1^) of the toxins were injected into the MS system by means of the LC system, omitting the chromatographic column, and using as carrier flow a 50:50 mixture of solvents A and B, to select the optimal MRM transitions for each compound. The adopted collision energies (CE) and fragmentation voltages are shown in Table 2.

Other equipment used included an EBA 20 centrifuge (Hettich, Tuttlingen, Germany) and an XcelVapTM evaporator (Horizon Technology Inc., Salem, NH, USA).

### 4.3. Samples

Ten seawater samples, collected from ten different points of the Mar Menor lagoon (Murcia, south-eastern Spain) in August 2019, were analyzed. The samples were differentiated into two types, those collected close to beaches (on foot) and those collected by boat from areas far from the coast. A 5 m column was used for sampling far from the coast, which allowed an integrated water sample from different depths to be analyzed. The beach samples were taken at a depth of 40 cm. All the samples were stored in square polyethylene containers of one-liter capacity at 4 °C until analysis, which was normally carried out within 48 h of arrival in the laboratory.

### 4.4. Analytical Procedure

Before analysis, the water samples were vacuum filtered through 0.45 µm nylon membrane filters (Agilent), and 12 mL of filtered samples were placed in a conical bottomed 15 mL Falcon tube. A mixture of 0.5 mL MeOH and 440 µL chloroform was injected rapidly into the sample, resulting in turbidity as a consequence of the dispersion of chloroform microdroplets. The ternary mixture was stirred manually for a few seconds and centrifuged at 855× *g* for 3 min. The sedimented phase was collected by micro syringe, and the solvent was evaporated by applying a pressure of 440 bar at 40 °C. The dry residue was reconstituted in 100 µL MeOH, and 15 µL were injected into the LC system. All samples were analyzed in duplicate.

Recovery studies were performed with two different samples at two levels of fortification: 10 and 50 ng·L^−1^ for all toxins except DTX (50 and 100 ng L^−1^). Three aliquots of each sample were analyzed at each concentration level.

## Figures and Tables

**Table 1:** Elution gradient program.

Time (min)	Solvent B (%)	Flow Rate (mL min^−1^)
0	25	0.3
3	60	0.3
8	60	0.3
8.5	75	0.3
15	75	0.3
15.5	75	0.4
20	85	0.4
20.5	95	0.5
26	95	0.5
31	25	0.5

**Table 2:** Liquid chromatography-triple quadrupole-tandem mass spectrometry (LC-QqQ-MS/MS) parameters for the lipophilic toxins analyzed.

Compound	Retention Time (min)	Multiple Reaction Monitoring Transition (*m/z*)	Fragmentation Voltage (V)	Collision Energy (V)
GYM	6.93	508.3 → 490.6 ^1^	200	40
13,19didesM	7.32	678 → 430 ^1^ 678 → 164 (194)	200 200	50 40
13desM	7.72	692 → 164 ^1^ 692 → 444 (44)	200 200	50 20
SPX20G	8.12	706 → 688 ^1^ 706 → 670 (33)	190 190	30 35
OA	13.8	827 → 723 ^1^ 827 → 809 (47)	190 190	55 45
DTX2	14.7	827 → 723 ^1^ 827 → 809 (40)	190 190	55 45
PTX2	14.8	881.5 → 539.3 ^1^ 881.5 → 837.5 (99)	230 230	60 70
AZA4	15.0	844 → 826 ^1^ 844 → 808 (12)	190 190	30 45
DTX1	17.5	841 → 737 ^1^ 841 → 823 (50)	190 190	45 55
AZA3	18.6	828 → 810 ^1^ 828 → 792 (13)	190 190	30 45
AZA5	19.0	844 → 826 ^1^ 844 → 808 (37)	180 180	30 40
AZA1	19.4	842 → 824 ^1^ 842 → 806 (17)	190 190	30 45
AZA2	20.0	856 → 838 ^1^ 856 → 820 (17)	190 190	30 45

^1^ Transition used for quantitation. Values in brackets mean relative abundance (expressed as a percentage) related to the product ion of the transition used for quantitation. GYM: gymnodimine; 13,19-didesM: 13,19-didesmethyl spirolide C; 13-desM: 13-desmethyl spirolide C; SPX20G: 20-spirolide G; OA: okadaic acid; DTX: dinophysitoxin; PTX2: pectenotoxin 2; AZA: azaspiracid.

**Table 3:** Central composite design (CCD) to study the relationship between the volumes of the three phases used in the dispersive liquid-liquid microextraction (DLLME) technique.

Assay	Point Type	Sample (mL)	MeOH (mL)	CHCl_3_ (µL)	Assay	Point Type	Sample (mL)	MeOH (mL)	CHCl_3_ (µL)
1	Cubic	6	2	200	11	Central	9	1.25	450
2	Cubic	12	0.5	700	12	Cubic	12	2	700
3	Cubic	12	2	200	13	Axial	9	1.25	575
4	Central	9	1.25	450	14	Axial	9	1.25	325
5	Cubic	12	0.5	200	15	Central	9	1.25	450
6	Cubic	6	0.5	200	16	Axial	9	1.625	450
7	Central	9	1.25	450	17	Axial	10.5	1.25	450
8	Cubic	6	2	700	18	Axial	7.5	1.25	450
9	Central	9	1.25	450	19	Axial	9	0.875	450
10	Cubic	6	0.5	700	20	central	9	1.25	450

**Table 4:** Analytical characteristics.

Compound	Linearity Range (ng L^−1^)	Limit of Detection (ng L^−1^)	Limit of Quantitation (ng L^−1^)	Relative Standard Deviation (%)
GYM	2.5–1000	0.7	2.3	0.1
13,19didesM	1.0–1000	0.3	1.0	3.1
13desM	1.0–1000	0.2	0.7	1.7
SPX20G	3.5–1000	1.0	3.3	0.9
OA	5.0–1500	1.4	4.7	0.8
DTX2	4.0–1000	1.1	3.7	1.9
PTX2	4.5–1000	1.1	3.7	2.3
AZA4	4.5–1000	1.3	4.3	5.1
DTX1	20–5000	5.7	19	7.5
AZA3	3.0–1000	0.9	3.0	2.1
AZA5	1.0–1000	0.2	0.7	1.8
AZA1	1.0–1000	0.3	1.0	1.0
AZA2	2.0–1000	0.6	2.0	3.6

GYM: gymnodimine; 13,19-didesM: 13,19-didesmethyl spirolide C; 13-desM: 13-desmethyl spirolide C; SPX20G: 20-spirolide G; OA: okadaic acid; DTX: dinophysitoxin; PTX2: pectenotoxin 2; AZA: azaspiracid.

**Table 5:** Recovery ^1^ studies in seawater samples.

Compound	Level (ng·L^−1^)	Sample 1	Sample 2	Compound	Level (ng·L^−1^)	Sample 1	Sample 2
GYM	10 50	100 98	112 102	AZA4	10 50	121 114	104 120
13,19didesM	10 50	89 92	90 94	DTX1	50 100	90 106	123 93
13desM	10 50	95 96	98 97	AZA3	10 50	101 106	113 106
SPX20G	10 50	82 119	114 121	AZA5	10 50	104 101	104 104
OA	10 50	111 99	109 96	AZA1	10 50	105 95	107 112
DTX2	10 50	90 105	118 90	AZA2	10 50	101 99	115 102
PTX2	10 50	96 102	112 112				

^1^ Mean value (*n* = 3). GYM: gymnodimine;13,19-didesM: 13,19-didesmethyl spirolide C; 13-desM: 13-desmethyl spirolide C; SPX20G: 20-spirolide G; OA: okadaic acid; DTX: dinophysitoxin; PTX2: pectenotoxin 2; AZA: azaspiracid.

**Table 6:** Comparison of the developed method with others previously published for the determination of toxins in seawater using liquid chromatography-mass spectrometry.

Sample Treatment				Limit of Detection Range (ng L^−1^)					Ref.
Technique	Sample Volume (mL)	Solvent Consumption	Time (min)	GYM	SPXs	OA and DTXs	PTXs	AZAs	
SPE	500	10 mL MeOH	532	1410	250	171–657.5	129	1237–1283	[22]
SPE	300	6 mL MeOH + 9 mL NH_4_OH/MeOH	338	25	23.5	34.2–128.9	60.6	8.5–82.4	[26]
SPE	200	9 mL MeOH	238	-	-	68	13	-	[27]
SPE	500	10 mL MeOH	520	-	-	0.3	0.5	0.002–0.003	[28]
DMSPE	50	1.5 mL NH_4_OH/AcN + 1.5 mL FA/AcN	-	0.03	0.03	0.2	-	0.03	[23]
IL-DLLME	10	0.5 mL AcN	3	-	-	1500	-	-	[31]
DLLME	12	0.5 mL MeOH + 0.44 mL CHCl_3_	5	0.7	0.2–1	1.1–5.7	1.1	0.2–1.3	This work

GYM: gymnodimine; SPX: spirolide; OA: okadaic acid; DTX: dinophysitoxin; PTX: pectenotoxin; AZA: azaspiracid. SPE: solid phase extraction; DMSPE: dispersive magnetic solid phase extraction; IL: ionic liquid; DLLME: dispersive liquid-liquid microextraction.

## Data Availability

Not applicable.
